# Supplementary material for: Heterogeneous macroeconomic factors’ effects on stocks across sizes, styles, and sectors in the South Korean market
Source: PLoS One. 2024 Apr 17;19(4):e0300393. doi: 10.1371/journal.pone.0300393 (PMC11023228; doi:10.1371/journal.pone.0300393)
Supplement: Attachment — Submitted filename: Reviewer Comments.docx [file pone.0300393.s001.docx]

This study elucidates the macroeconomic variables that have a significant impact on stock volatility, using the Korean market as an example.The article also analyses the relationship between macroeconomic variables and stock return volatility indices.The research seems interesting, but the following questions still need to be further considered:

1. Formatting problems - Figure 1-8 is somewhat blurry and unclear, and the letters are not clearly visible.
2. Research paradigm - The article is very rich in research methods, including a series of models and formulas, can you write the background, advantages and limitations of the applied methods clearly, so as to facilitate the readers to read and study.
3. Introduction - The repetition of certain ideas or information within the text could be condensed to avoid redundancy. For example, the discussion about the impact of macroeconomic indicators on different stock sizes and sectors could be streamlined for clearer presentation.While the introduction states the significance of the study in analyzing the effects of macroeconomic variables on stock volatility, it could more explicitly define the specific research objectives and identify the existing gaps in the literature that this study intends to address. Clearly stating what this research aims to contribute to the existing knowledge base would enhance the paper's value.
4. Literature review - The literature review does not sufficiently highlight the relevance to the Korean stock market and may need to add more literature on the Korean stock market.
5. Research design - This study investigates the relationship between macroeconomic variables and stock volatility based on capital size, style, and sector in South Korea through an analysis of S&P South Korea BMI LargeMidCap, SmallCap, Growth, and Value, and Sectors Index values from January 2009 to September 2022. The article has no test for endogeneity of causality.This part of the article may need refining.
6. The article doesn't write about the limitations of the study.
7. English language logic and expression need to be improved
